# A dinuclear copper complex: bis­(μ-4-amino­benzoato)bis­[aqua(1,10-phenanthroline)copper(II)] dichloride bis(4-amino­benzoic acid) dihydrate

**DOI:** 10.1107/S1600536808023647

**Published:** 2008-07-31

**Authors:** Miao-Ling Huang, Qing-Fan Xie, Jing-Chun Xian, Yan-Min Chen, Zi-Qiao Zhou

**Affiliations:** a Department of Chemistry and Science of life, Quanzhou Normal University, Fujian 362000, People’s Republic of China

## Abstract

The title complex, [Cu_2_(C_7_H_6_NO_2_)_2_(C_12_H_8_N_2_)_2_(H_2_O)_2_]·2C_7_H_7_NO_2_·2H_2_O, consists of a dinuclear [Cu_2_(C_7_H_6_NO_2_)_2_(C_12_H_8_N_2_)_2_(H_2_O)_2_]^2+^ cation, two Cl^−^ anions, two 4-amino­benzoic acid mol­ecules and two disordered water mol­ecules (site occupancy factors 0.5). The Cu(II) ion adopts a distorted square-pyramidal geometry formed by two N atoms from the 1,10-phenanthroline ligand and two O atoms of the two 4-amino­benzoic acid ligands and one water O atom. The Cu⋯Cu separation is 3.109 (2) Å. A twofold axis passes through the mid-point of the Cu⋯Cu vector.

## Related literature

For related literature, see: Lo *et al.* (2000 ▶); Zoroddu *et al.* (1996 ▶); Rao *et al.* (2004 ▶); Müller *et al.* (2003 ▶).
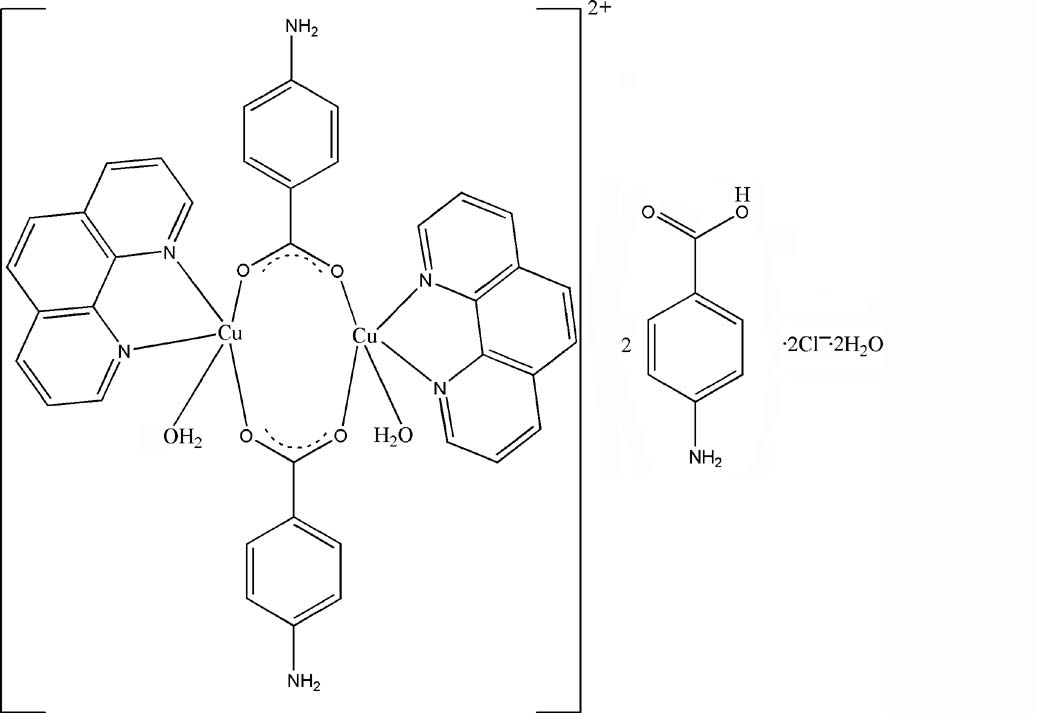


## Experimental

### 

#### Crystal data

[Cu_2_(C_7_H_6_NO_2_)_2_(C_12_H_8_N_2_)_2_(H_2_O)_2_]·2C_7_H_7_NO_2_·2H_2_O
*M* *_r_* = 1174.96
Monoclinic, 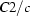

*a* = 25.748 (2) Å
*b* = 10.0988 (8) Å
*c* = 20.9156 (17) Å
β = 110.3070 (10)°
*V* = 5100.5 (7) Å^3^
*Z* = 4
Mo *K*α radiation
μ = 1.01 mm^−1^
*T* = 291 (2) K
0.49 × 0.40 × 0.37 mm

#### Data collection

Bruker SMART CCD area-detector diffractometer
Absorption correction: multi-scan (*SADABS*; Bruker, 2001 ▶) *T*_min_ = 0.638, *T*_max_ = 0.708
18514 measured reflections
4744 independent reflections
3886 reflections with *I* > 2σ(*I*)
*R*_int_ = 0.030

#### Refinement

*R*[*F* ^2^ > 2σ(*F* ^2^)] = 0.032
*wR*(*F* ^2^) = 0.091
*S* = 1.02
4744 reflections
352 parameters
H-atom parameters constrained
Δρ_max_ = 0.49 e Å^−3^
Δρ_min_ = −0.31 e Å^−3^

### 

Data collection: *SMART* (Bruker, 2001 ▶); cell refinement: *SAINT* (Bruker, 2001 ▶); data reduction: *SAINT*; program(s) used to solve structure: *SHELXS97* (Sheldrick, 2008 ▶); program(s) used to refine structure: *SHELXL97* (Sheldrick, 2008 ▶); molecular graphics: *ORTEP-3 for Windows* (Farrugia, 1997 ▶); software used to prepare material for publication: *SHELXTL* (Sheldrick, 2008 ▶).

## Supplementary Material

Crystal structure: contains datablocks global, I. DOI: 10.1107/S1600536808023647/pv2085sup1.cif

Structure factors: contains datablocks I. DOI: 10.1107/S1600536808023647/pv2085Isup2.hkl

Additional supplementary materials:  crystallographic information; 3D view; checkCIF report

## Figures and Tables

**Table 1:** Hydrogen-bond geometry (Å, °)

*D*—H⋯*A*	*D*—H	H⋯*A*	*D*⋯*A*	*D*—H⋯*A*
O1—H2*W*⋯Cl1^i^	0.85	2.71	3.549 (7)	170
O1—H1*W*⋯O1^ii^	0.88	2.32	2.938 (11)	127
O2—H4*W*⋯O1	0.82	1.80	2.477 (8)	138
O2—H3*W*⋯N4	0.83	2.30	3.081 (5)	157
O3—H5*W*⋯Cl1	0.83	2.32	3.1259 (17)	163
O3—H6*W*⋯O6	0.84	1.90	2.737 (2)	179
N3—H3*A*⋯Cl1^iii^	0.86	2.68	3.475 (3)	154
N3—H3*B*⋯O2^iv^	0.86	2.07	2.898 (5)	161
N4—H4*A*⋯Cl1^v^	0.86	2.71	3.512 (3)	155
N4—H4*B*⋯Cl1^i^	0.86	2.59	3.433 (3)	167

Symmetry codes: (i) 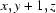; (ii) 
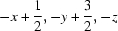
; (iii) 
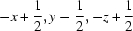
; (iv) 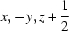; (v) 
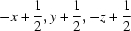
.

**Fig. 1.**
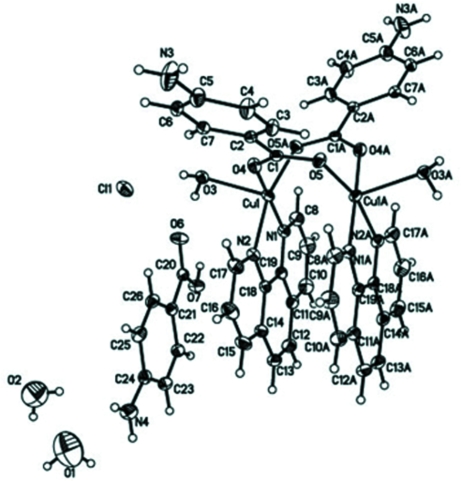
The ORTEP-3 (Farrugia, 1997) drawing of the title compound. Displacement ellipsoids are drawn at 30% probability level. Symmetry code for letter "A" in the atomic symbols: -x, y, -z+1/2.

**Fig. 2.**
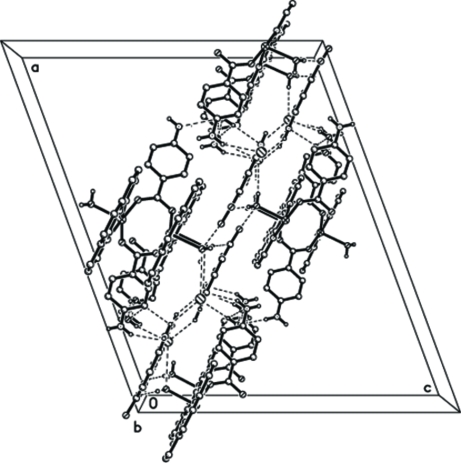
Projection of the unit cell showing the three-dimensional structure formed by H-bonding interaction of the compound (I).

## supplementary crystallographic information

### Comment

During the last three decades, copper complexes have received much attention because of their interesting interactions with biological ligands to generate stable mixed coordinated complexes, which play a key role in life processes such as enzymatic catalysis, storage and conveyance of the matter, transfer of copper ions (Müller, *et al.*, 2003; Rao, *et al.*, 2004; Lo, *et al.*, 2000). 4-Aminobenzoic acid, an important part of folic acid, is a constituent of the vitamin B complex and is found in animal and plant tissues, and has been shown to be a growth factor in certain microorganisms, particularly Enterococci and Lactobacilli (Zoroddu *et al.*, 1996). In order to extend further the study of 4-aminobenzoic acid ligand coordinated to copper ion, we have synthesized the title complex, (I) and determined its crystal structure by X-ray diffraction which is presented in this article.

The molecular structure and crystal packing diagram of the title compound are presented in Figs. 1 and 2, respectively. The structure is composed of a dimeric [Cu_2_(C_7_H_6_NO_2_)_2_(C_12_H_8_N_2_)_2_(H_2_O)_2_]_2_^+^ cation with two five-coordinated Cu(II) ions linked by two oxygen atoms of 4-aminobenzoic acid, two Cl^-^ anions, two 4-aminobenzoic acid molecules and two disordered water molecules lying over four sites with 0.5 occupancy factors each. The Cu(II) ion has a distorted square-pyramidal geometry with two N atoms of 1,10-phenanthroline ligand and two O atoms of two 4-aminobenzoic acid ligands occupying basal sites and the apical position being occupied by an O atom of H_2_O. The benzoic acid molecules not coordinated to Cu are hydrogen bonded to Cl^-^ ions by amino H-atoms and to water of coordination by hydroxyl H-atoms (details are given in Table 1). As a matter of fact, the complex molecules, Cl^-^ anions, 4-aminobenzoic acid molecules and water molecules are linked by a network of O—H···O, N—H···O and O—H···Cl hydrogen bonds into a three-dimensional supramolecular structure. In the complex, the two 1,10-phenanthroline ligands are stacked with their centroids separated by 3.661 (2) Å indicating significant π-π interactions.

### Experimental

An aqueous solution (5 ml) of CuCl_2_.3H_2_O (1 mmol) was added slowly to a mixed solution of 4-aminobenzoic acid (1.5 mmol) in H_2_O (5 ml) and 1,10-phenanthroline (1 mmol) in ethanol (95%, 5 ml). After refluxing for 3 h, the mixture was filtered off while hot. The dark-green single crystals suitable for X-ray analysis were obtained by slow evaporation of the above filtrate at room temperature after a week.

### Refinement

The occupancy factors of water molecules not involved in coordination refined close to 0.5 values at the initial stages. Hence, site occupancy factors for both water molecules were fixed at 0.5. H-atoms bonded to water molecules were taken from a difference Fourier map and were fixed at those positions during the refinements with *U*_iso_(H) = 1.5*U*_eq_(O). H atoms bonded to C, N and hydroxyl O atoms were placed geometrically and treated as riding, with distances C—H = 0.93, N—H = 0.86 and O—H = 0.82 Å and *U*_iso_(H) = 1.2*U*_eq_(C and N) and *U*_iso_(H) = 1.5*U*_eq_(O).

### Crystal data

**Table:**

[Cu_2_(C_7_H_6_NO_2_)_2_(C_12_H_8_N_2_)_2_(H_2_O)_2_]·2C_7_H_7_NO_2_·2H_2_O	*F*_000_ = 2416
*M**_r_* = 1174.96	*D*_x_ = 1.530 Mg m^−^^3^
Monoclinic, *C*2/*c*	Mo *K*α radiation λ = 0.71073 Å
Hall symbol: -C 2yc	Cell parameters from 5516 reflections
*a* = 25.748 (2) Å	θ = 2.3–24.7º
*b* = 10.0988 (8) Å	µ = 1.01 mm^−^^1^
*c* = 20.9156 (17) Å	*T* = 291 (2) K
β = 110.3070 (10)º	Block, blue
*V* = 5100.5 (7) Å^3^	0.49 × 0.40 × 0.37 mm
*Z* = 4	

### Data collection

**Table:**

Bruker SMART CCD area-detector diffractometer	4744 independent reflections
Radiation source: fine-focus sealed tube	3886 reflections with *I* > 2σ(*I*)
Monochromator: graphite	*R*_int_ = 0.030
*T* = 291(2) K	θ_max_ = 25.5º
φ & ω scans	θ_min_ = 2.3º
Absorption correction: multi-scan(SADABS; Bruker, 2001)	*h* = −31→31
*T*_min_ = 0.638, *T*_max_ = 0.708	*k* = −12→12
18514 measured reflections	*l* = −24→25

### Refinement

**Table:**

Refinement on *F*^2^	Secondary atom site location: difference Fourier map
Least-squares matrix: full	Hydrogen site location: inferred from neighbouring sites
*R*[*F*^2^ > 2σ(*F*^2^)] = 0.032	H-atom parameters constrained
*wR*(*F*^2^) = 0.091	*w* = 1/[σ^2^(*F*_o_^2^) + (0.0477*P*)^2^ + 4.1583*P*] where *P* = (*F*_o_^2^ + 2*F*_c_^2^)/3
*S* = 1.02	(Δ/σ)_max_ < 0.001
4744 reflections	Δρ_max_ = 0.49 e Å^−^^3^
352 parameters	Δρ_min_ = −0.31 e Å^−^^3^
Primary atom site location: structure-invariant direct methods	Extinction correction: none

### Special details

**Table:**

Experimental. Yield 81%. IR(KBr): 3409(*s*), 3190(w), 3057(w), 2921(*m*), 2857(w), 1734(w), 1671(*s*), 1630(*s*), 1600(*versus*), 1550(*s*), 1516(*s*), 1494(w), 1418(*m*), 1389(*versus*), 1344(w), 1312(*m*), 1267(*s*), 1222(vw), 1178(*s*), 1146(*m*), 1108(*m*), 1046(w), 854(*s*), 844(*s*), 786(*m*), 719(*s*), 702(w), 643(*m*), 609(*m*), 511(*m*), 466(w), 430(w).
Geometry. All e.s.d.'s (except the e.s.d. in the dihedral angle between two l.s. planes)are estimated using the full covariance matrix. The cell e.s.d.'s are takeninto account individually in the estimation of e.s.d.'s in distances, anglesand torsion angles; correlations between e.s.d.'s in cell parameters are onlyused when they are defined by crystal symmetry. An approximate (isotropic)treatment of cell e.s.d.'s is used for estimating e.s.d.'s involving l.s. planes.
Refinement. Refinement of *F*^2^ against ALL reflections. The weighted *R*-factor *wR* andgoodness of fit *S* are based on *F*^2^, conventional *R*-factors *R* are basedon *F*, with *F* set to zero for negative *F*^2^. The threshold expression of*F*^2^ > σ(*F*^2^) is used only for calculating *R*-factors(gt) *etc*. and isnot relevant to the choice of reflections for refinement. *R*-factors basedon *F*^2^ are statistically about twice as large as those based on *F*, and *R*-factors based on ALL data will be even larger.

### Fractional atomic coordinates and isotropic or equivalent isotropic displacement parameters (Å^2^)

**Table:**

	*x*	*y*	*z*	*U*_iso_*/*U*_eq_	Occ. (<1)
Cu1	0.018177 (11)	−0.03851 (3)	0.186473 (14)	0.03323 (10)	
Cl1	0.18713 (3)	−0.11806 (7)	0.16975 (4)	0.0568 (2)	
O1	0.2421 (2)	0.7015 (7)	0.0622 (3)	0.117 (2)	0.50
H2W	0.2255	0.7481	0.0828	0.176*	0.50
H1W	0.2651	0.7497	0.0492	0.176*	0.50
O2	0.29142 (16)	0.4893 (4)	0.10037 (19)	0.0606 (10)	0.50
H4W	0.2905	0.5658	0.0865	0.091*	0.50
H3W	0.2722	0.4821	0.1246	0.091*	0.50
O4	0.06914 (7)	−0.15420 (17)	0.25246 (9)	0.0441 (4)	
O3	0.05793 (6)	−0.10211 (15)	0.10938 (8)	0.0389 (4)	
H5W	0.0910	−0.1233	0.1281	0.058*	
H6W	0.0554	−0.0384	0.0830	0.058*	
O5	0.04104 (7)	−0.16729 (16)	0.34272 (8)	0.0416 (4)	
O6	0.05048 (8)	0.10828 (17)	0.02418 (10)	0.0561 (5)	
O7	0.00758 (7)	0.29085 (17)	−0.02501 (9)	0.0500 (4)	
N1	−0.03250 (8)	0.10025 (19)	0.12729 (10)	0.0363 (4)	
N2	0.06502 (8)	0.12213 (19)	0.22432 (10)	0.0367 (4)	
N3	0.26796 (11)	−0.4815 (3)	0.45449 (15)	0.0914 (11)	
H3A	0.2899	−0.5052	0.4336	0.110*	
H3B	0.2751	−0.5037	0.4964	0.110*	
N4	0.22137 (10)	0.5522 (2)	0.18975 (12)	0.0609 (7)	
H4A	0.2511	0.5160	0.2169	0.073*	
H4B	0.2188	0.6370	0.1867	0.073*	
C1	0.07507 (9)	−0.1908 (2)	0.31242 (12)	0.0338 (5)	
C2	0.12577 (9)	−0.2661 (2)	0.35019 (11)	0.0323 (5)	
C3	0.13794 (10)	−0.2994 (3)	0.41806 (12)	0.0441 (6)	
H3D	0.1137	−0.2744	0.4400	0.053*	
C4	0.18489 (11)	−0.3682 (3)	0.45348 (13)	0.0539 (7)	
H4	0.1925	−0.3883	0.4992	0.065*	
C5	0.22162 (11)	−0.4085 (3)	0.42110 (14)	0.0515 (7)	
C6	0.21002 (10)	−0.3726 (2)	0.35347 (13)	0.0411 (6)	
H6	0.2343	−0.3967	0.3314	0.049*	
C7	0.16336 (10)	−0.3024 (2)	0.31893 (12)	0.0363 (5)	
H7A	0.1566	−0.2785	0.2738	0.044*	
C8	−0.08038 (11)	0.0855 (3)	0.07639 (13)	0.0480 (6)	
H8	−0.0935	0.0003	0.0632	0.058*	
C9	−0.11160 (12)	0.1930 (3)	0.04215 (14)	0.0582 (8)	
H9	−0.1447	0.1792	0.0062	0.070*	
C10	−0.09364 (12)	0.3183 (3)	0.06132 (14)	0.0556 (7)	
H10	−0.1150	0.3903	0.0396	0.067*	
C11	−0.04277 (11)	0.3387 (2)	0.11407 (13)	0.0438 (6)	
C12	−0.01910 (14)	0.4660 (3)	0.13788 (16)	0.0540 (7)	
H12	−0.0383	0.5422	0.1183	0.065*	
C13	0.03050 (14)	0.4771 (3)	0.18812 (16)	0.0553 (8)	
H13	0.0448	0.5608	0.2025	0.066*	
C14	0.06162 (11)	0.3625 (2)	0.21979 (13)	0.0438 (6)	
C15	0.11401 (13)	0.3645 (3)	0.27098 (15)	0.0593 (8)	
H15	0.1308	0.4450	0.2876	0.071*	
C16	0.14047 (12)	0.2497 (3)	0.29647 (15)	0.0612 (8)	
H16	0.1754	0.2516	0.3300	0.073*	
C17	0.11497 (11)	0.1290 (3)	0.27211 (14)	0.0497 (7)	
H17	0.1335	0.0508	0.2898	0.060*	
C18	0.03912 (10)	0.2371 (2)	0.19804 (12)	0.0358 (5)	
C19	−0.01349 (10)	0.2252 (2)	0.14510 (11)	0.0349 (5)	
C20	0.04919 (10)	0.2283 (2)	0.02199 (12)	0.0386 (6)	
C21	0.09205 (10)	0.3146 (2)	0.06716 (12)	0.0364 (5)	
C22	0.08804 (10)	0.4523 (2)	0.06392 (12)	0.0393 (5)	
H22	0.0565	0.4916	0.0335	0.047*	
C23	0.13002 (11)	0.5308 (2)	0.10500 (13)	0.0440 (6)	
H23	0.1265	0.6224	0.1020	0.053*	
C24	0.17799 (10)	0.4743 (3)	0.15117 (12)	0.0422 (6)	
C25	0.18133 (11)	0.3366 (3)	0.15532 (13)	0.0486 (6)	
H25	0.2125	0.2971	0.1865	0.058*	
C26	0.13959 (11)	0.2583 (3)	0.11425 (12)	0.0436 (6)	
H26	0.1429	0.1666	0.1177	0.052*	

### Atomic displacement parameters (Å^2^)

**Table:**

	*U*^11^	*U*^22^	*U*^33^	*U*^12^	*U*^13^	*U*^23^
Cu1	0.02996 (16)	0.03028 (16)	0.03771 (17)	−0.00293 (11)	0.00953 (12)	0.00503 (12)
Cl1	0.0428 (4)	0.0556 (4)	0.0662 (5)	−0.0092 (3)	0.0117 (3)	0.0103 (3)
O1	0.077 (4)	0.185 (6)	0.079 (4)	−0.016 (4)	0.014 (3)	0.018 (4)
O2	0.063 (3)	0.070 (3)	0.045 (2)	−0.003 (2)	0.0136 (19)	−0.0081 (19)
O4	0.0407 (9)	0.0462 (10)	0.0469 (10)	0.0072 (8)	0.0169 (8)	0.0165 (8)
O3	0.0351 (9)	0.0391 (9)	0.0410 (9)	0.0007 (7)	0.0113 (7)	0.0082 (7)
O5	0.0380 (9)	0.0419 (9)	0.0465 (10)	0.0118 (7)	0.0166 (8)	0.0016 (8)
O6	0.0755 (14)	0.0345 (10)	0.0529 (11)	−0.0070 (9)	0.0154 (10)	0.0066 (8)
O7	0.0495 (10)	0.0397 (10)	0.0520 (11)	0.0027 (8)	0.0062 (9)	0.0031 (8)
N1	0.0353 (10)	0.0365 (11)	0.0361 (11)	−0.0009 (9)	0.0111 (9)	0.0023 (9)
N2	0.0331 (10)	0.0374 (11)	0.0373 (11)	−0.0048 (9)	0.0095 (9)	0.0034 (9)
N3	0.0673 (18)	0.142 (3)	0.0690 (18)	0.0646 (19)	0.0284 (15)	0.0421 (19)
N4	0.0495 (14)	0.0600 (16)	0.0599 (15)	−0.0008 (12)	0.0019 (12)	−0.0113 (12)
C1	0.0342 (12)	0.0238 (11)	0.0429 (13)	−0.0006 (9)	0.0128 (11)	0.0010 (10)
C2	0.0327 (12)	0.0260 (11)	0.0388 (12)	0.0016 (9)	0.0134 (10)	0.0024 (9)
C3	0.0414 (14)	0.0528 (16)	0.0435 (14)	0.0109 (12)	0.0217 (12)	0.0064 (12)
C4	0.0495 (16)	0.076 (2)	0.0372 (14)	0.0200 (14)	0.0157 (12)	0.0175 (13)
C5	0.0407 (14)	0.0609 (17)	0.0525 (16)	0.0171 (13)	0.0158 (12)	0.0143 (14)
C6	0.0351 (13)	0.0425 (14)	0.0504 (15)	0.0054 (11)	0.0209 (11)	0.0032 (11)
C7	0.0394 (13)	0.0324 (12)	0.0401 (13)	0.0003 (10)	0.0178 (11)	0.0041 (10)
C8	0.0414 (14)	0.0509 (16)	0.0456 (15)	−0.0030 (12)	0.0074 (12)	0.0005 (12)
C9	0.0472 (16)	0.068 (2)	0.0487 (16)	0.0102 (15)	0.0031 (13)	0.0110 (15)
C10	0.0576 (17)	0.0569 (18)	0.0518 (16)	0.0170 (14)	0.0181 (14)	0.0209 (14)
C11	0.0547 (16)	0.0399 (14)	0.0438 (14)	0.0092 (12)	0.0261 (13)	0.0113 (11)
C12	0.074 (2)	0.0357 (14)	0.0630 (18)	0.0076 (14)	0.0373 (17)	0.0108 (13)
C13	0.082 (2)	0.0323 (14)	0.068 (2)	−0.0069 (14)	0.0465 (19)	−0.0047 (13)
C14	0.0550 (16)	0.0373 (13)	0.0464 (14)	−0.0112 (12)	0.0267 (13)	−0.0077 (11)
C15	0.068 (2)	0.0518 (17)	0.0625 (18)	−0.0276 (16)	0.0289 (16)	−0.0162 (15)
C16	0.0493 (17)	0.070 (2)	0.0561 (18)	−0.0207 (15)	0.0077 (14)	−0.0072 (16)
C17	0.0398 (14)	0.0534 (16)	0.0495 (15)	−0.0067 (12)	0.0072 (12)	0.0056 (13)
C18	0.0419 (13)	0.0352 (12)	0.0362 (12)	−0.0046 (10)	0.0210 (11)	0.0007 (10)
C19	0.0412 (13)	0.0344 (12)	0.0332 (12)	−0.0002 (10)	0.0181 (10)	0.0034 (10)
C20	0.0483 (15)	0.0356 (14)	0.0384 (13)	−0.0007 (11)	0.0234 (12)	0.0034 (10)
C21	0.0414 (13)	0.0370 (13)	0.0343 (12)	0.0019 (11)	0.0176 (10)	0.0034 (10)
C22	0.0395 (13)	0.0389 (13)	0.0376 (13)	0.0043 (11)	0.0111 (11)	0.0055 (11)
C23	0.0498 (15)	0.0359 (14)	0.0452 (14)	0.0039 (12)	0.0153 (12)	0.0022 (11)
C24	0.0419 (14)	0.0496 (16)	0.0362 (13)	−0.0001 (12)	0.0148 (11)	−0.0044 (11)
C25	0.0442 (15)	0.0526 (16)	0.0441 (15)	0.0140 (13)	0.0089 (12)	0.0028 (12)
C26	0.0507 (15)	0.0367 (13)	0.0443 (14)	0.0103 (12)	0.0176 (12)	0.0045 (11)

### Geometric parameters (Å, °)

**Table:**

Cu1—O4	1.9326 (16)	C6—C7	1.366 (3)
Cu1—O5^i^	1.9346 (16)	C6—H6	0.9300
Cu1—N2	2.0125 (19)	C7—H7A	0.9300
Cu1—N1	2.0192 (19)	C8—C9	1.393 (4)
Cu1—O3	2.2815 (16)	C8—H8	0.9300
O1—H2W	0.8467	C9—C10	1.358 (4)
O1—H1W	0.8810	C9—H9	0.9300
O2—H4W	0.8235	C10—C11	1.404 (4)
O2—H3W	0.8250	C10—H10	0.9300
O4—C1	1.265 (3)	C11—C19	1.401 (3)
O3—H5W	0.8317	C11—C12	1.436 (4)
O3—H6W	0.8354	C12—C13	1.348 (4)
O5—C1	1.269 (3)	C12—H12	0.9300
O5—Cu1^i^	1.9346 (16)	C13—C14	1.432 (4)
O6—C20	1.213 (3)	C13—H13	0.9300
O7—C20	1.335 (3)	C14—C18	1.401 (3)
N1—C8	1.328 (3)	C14—C15	1.401 (4)
N1—C19	1.358 (3)	C15—C16	1.356 (4)
N2—C17	1.329 (3)	C15—H15	0.9300
N2—C18	1.357 (3)	C16—C17	1.395 (4)
N3—C5	1.370 (3)	C16—H16	0.9300
N3—H3A	0.8600	C17—H17	0.9300
N3—H3B	0.8600	C18—C19	1.426 (3)
N4—C24	1.375 (3)	C20—C21	1.465 (3)
N4—H4A	0.8600	C21—C22	1.394 (3)
N4—H4B	0.8600	C21—C26	1.398 (3)
C1—C2	1.480 (3)	C22—C23	1.375 (3)
C2—C3	1.385 (3)	C22—H22	0.9300
C2—C7	1.392 (3)	C23—C24	1.399 (3)
C3—C4	1.368 (3)	C23—H23	0.9300
C3—H3D	0.9300	C24—C25	1.394 (4)
C4—C5	1.401 (4)	C25—C26	1.370 (4)
C4—H4	0.9300	C25—H25	0.9300
C5—C6	1.389 (4)	C26—H26	0.9300
			
O4—Cu1—O5^i^	94.84 (7)	C10—C9—H9	120.1
O4—Cu1—N2	92.34 (8)	C8—C9—H9	120.1
O5^i^—Cu1—N2	164.82 (8)	C9—C10—C11	119.8 (3)
O4—Cu1—N1	172.52 (8)	C9—C10—H10	120.1
O5^i^—Cu1—N1	90.13 (7)	C11—C10—H10	120.1
N2—Cu1—N1	81.56 (8)	C19—C11—C10	116.7 (2)
O4—Cu1—O3	88.54 (6)	C19—C11—C12	118.4 (2)
O5^i^—Cu1—O3	94.90 (6)	C10—C11—C12	124.9 (3)
N2—Cu1—O3	98.65 (7)	C13—C12—C11	121.2 (3)
N1—Cu1—O3	96.61 (7)	C13—C12—H12	119.4
H2W—O1—H1W	111.3	C11—C12—H12	119.4
H4W—O2—H3W	110.4	C12—C13—C14	121.3 (3)
C1—O4—Cu1	134.68 (15)	C12—C13—H13	119.3
Cu1—O3—H5W	112.1	C14—C13—H13	119.3
Cu1—O3—H6W	107.4	C18—C14—C15	116.2 (3)
H5W—O3—H6W	110.0	C18—C14—C13	118.5 (2)
C1—O5—Cu1^i^	124.96 (15)	C15—C14—C13	125.3 (3)
C8—N1—C19	118.0 (2)	C16—C15—C14	120.5 (3)
C8—N1—Cu1	129.48 (18)	C16—C15—H15	119.8
C19—N1—Cu1	112.46 (15)	C14—C15—H15	119.8
C17—N2—C18	118.1 (2)	C15—C16—C17	119.7 (3)
C17—N2—Cu1	128.99 (18)	C15—C16—H16	120.2
C18—N2—Cu1	112.80 (15)	C17—C16—H16	120.2
C5—N3—H3A	120.0	N2—C17—C16	122.0 (3)
C5—N3—H3B	120.0	N2—C17—H17	119.0
H3A—N3—H3B	120.0	C16—C17—H17	119.0
C24—N4—H4A	120.0	N2—C18—C14	123.5 (2)
C24—N4—H4B	120.0	N2—C18—C19	116.3 (2)
H4A—N4—H4B	120.0	C14—C18—C19	120.2 (2)
O4—C1—O5	125.0 (2)	N1—C19—C11	123.2 (2)
O4—C1—C2	117.4 (2)	N1—C19—C18	116.5 (2)
O5—C1—C2	117.6 (2)	C11—C19—C18	120.3 (2)
C3—C2—C7	118.1 (2)	O6—C20—O7	120.3 (2)
C3—C2—C1	121.0 (2)	O6—C20—C21	124.4 (2)
C7—C2—C1	120.9 (2)	O7—C20—C21	115.2 (2)
C4—C3—C2	121.4 (2)	C22—C21—C26	118.2 (2)
C4—C3—H3D	119.3	C22—C21—C20	122.4 (2)
C2—C3—H3D	119.3	C26—C21—C20	119.4 (2)
C3—C4—C5	120.2 (2)	C23—C22—C21	121.1 (2)
C3—C4—H4	119.9	C23—C22—H22	119.5
C5—C4—H4	119.9	C21—C22—H22	119.5
N3—C5—C6	120.1 (2)	C22—C23—C24	120.7 (2)
N3—C5—C4	121.6 (3)	C22—C23—H23	119.6
C6—C5—C4	118.3 (2)	C24—C23—H23	119.6
C7—C6—C5	120.9 (2)	N4—C24—C25	120.9 (2)
C7—C6—H6	119.6	N4—C24—C23	121.0 (2)
C5—C6—H6	119.6	C25—C24—C23	118.1 (2)
C6—C7—C2	121.0 (2)	C26—C25—C24	121.2 (2)
C6—C7—H7A	119.5	C26—C25—H25	119.4
C2—C7—H7A	119.5	C24—C25—H25	119.4
N1—C8—C9	122.3 (3)	C25—C26—C21	120.7 (2)
N1—C8—H8	118.9	C25—C26—H26	119.6
C9—C8—H8	118.9	C21—C26—H26	119.6
C10—C9—C8	119.9 (3)		
			
O5^i^—Cu1—O4—C1	−79.3 (2)	C11—C12—C13—C14	0.0 (4)
N2—Cu1—O4—C1	87.3 (2)	C12—C13—C14—C18	−0.8 (4)
O3—Cu1—O4—C1	−174.1 (2)	C12—C13—C14—C15	178.2 (3)
O5^i^—Cu1—N1—C8	−16.4 (2)	C18—C14—C15—C16	0.5 (4)
N2—Cu1—N1—C8	176.4 (2)	C13—C14—C15—C16	−178.5 (3)
O3—Cu1—N1—C8	78.6 (2)	C14—C15—C16—C17	−0.8 (5)
O5^i^—Cu1—N1—C19	162.21 (16)	C18—N2—C17—C16	1.4 (4)
N2—Cu1—N1—C19	−5.03 (15)	Cu1—N2—C17—C16	−175.2 (2)
O3—Cu1—N1—C19	−102.84 (15)	C15—C16—C17—N2	−0.2 (5)
O4—Cu1—N2—C17	6.6 (2)	C17—N2—C18—C14	−1.7 (3)
O5^i^—Cu1—N2—C17	124.8 (3)	Cu1—N2—C18—C14	175.41 (18)
N1—Cu1—N2—C17	−177.8 (2)	C17—N2—C18—C19	177.7 (2)
O3—Cu1—N2—C17	−82.3 (2)	Cu1—N2—C18—C19	−5.2 (2)
O4—Cu1—N2—C18	−170.09 (16)	C15—C14—C18—N2	0.7 (4)
O5^i^—Cu1—N2—C18	−51.9 (4)	C13—C14—C18—N2	179.8 (2)
N1—Cu1—N2—C18	5.57 (15)	C15—C14—C18—C19	−178.6 (2)
O3—Cu1—N2—C18	101.03 (15)	C13—C14—C18—C19	0.5 (3)
Cu1—O4—C1—O5	9.1 (4)	C8—N1—C19—C11	2.3 (3)
Cu1—O4—C1—C2	−170.59 (15)	Cu1—N1—C19—C11	−176.46 (18)
Cu1^i^—O5—C1—O4	−14.8 (3)	C8—N1—C19—C18	−177.5 (2)
Cu1^i^—O5—C1—C2	164.80 (15)	Cu1—N1—C19—C18	3.7 (2)
O4—C1—C2—C3	174.1 (2)	C10—C11—C19—N1	−1.2 (4)
O5—C1—C2—C3	−5.6 (3)	C12—C11—C19—N1	178.9 (2)
O4—C1—C2—C7	−4.2 (3)	C10—C11—C19—C18	178.6 (2)
O5—C1—C2—C7	176.1 (2)	C12—C11—C19—C18	−1.3 (3)
C7—C2—C3—C4	−1.3 (4)	N2—C18—C19—N1	1.0 (3)
C1—C2—C3—C4	−179.6 (2)	C14—C18—C19—N1	−179.6 (2)
C2—C3—C4—C5	−1.0 (4)	N2—C18—C19—C11	−178.8 (2)
C3—C4—C5—N3	−177.7 (3)	C14—C18—C19—C11	0.6 (3)
C3—C4—C5—C6	2.3 (4)	O6—C20—C21—C22	−178.2 (2)
N3—C5—C6—C7	178.5 (3)	O7—C20—C21—C22	3.6 (3)
C4—C5—C6—C7	−1.5 (4)	O6—C20—C21—C26	3.5 (4)
C5—C6—C7—C2	−0.7 (4)	O7—C20—C21—C26	−174.7 (2)
C3—C2—C7—C6	2.1 (4)	C26—C21—C22—C23	1.1 (4)
C1—C2—C7—C6	−179.6 (2)	C20—C21—C22—C23	−177.2 (2)
C19—N1—C8—C9	−1.2 (4)	C21—C22—C23—C24	0.1 (4)
Cu1—N1—C8—C9	177.4 (2)	C22—C23—C24—N4	176.5 (2)
N1—C8—C9—C10	−1.0 (4)	C22—C23—C24—C25	−1.4 (4)
C8—C9—C10—C11	2.1 (4)	N4—C24—C25—C26	−176.2 (2)
C9—C10—C11—C19	−1.1 (4)	C23—C24—C25—C26	1.7 (4)
C9—C10—C11—C12	178.8 (3)	C24—C25—C26—C21	−0.5 (4)
C19—C11—C12—C13	1.0 (4)	C22—C21—C26—C25	−0.9 (4)
C10—C11—C12—C13	−178.8 (3)	C20—C21—C26—C25	177.5 (2)

Symmetry codes: (i) −*x*, *y*, −*z*+1/2.

### Hydrogen-bond geometry (Å, °)

**Table:**

*D*—H···*A*	*D*—H	H···*A*	*D*···*A*	*D*—H···*A*
O1—H2W···Cl1^ii^	0.85	2.71	3.549 (7)	170
O1—H1W···O1^iii^	0.88	2.32	2.938 (11)	127
O2—H4W···O1	0.82	1.80	2.477 (8)	138
O2—H3W···N4	0.83	2.30	3.081 (5)	157
O3—H5W···Cl1	0.83	2.32	3.1259 (17)	163
O3—H6W···O6	0.84	1.90	2.737 (2)	179
N3—H3A···Cl1^iv^	0.86	2.68	3.475 (3)	154
N3—H3B···O2^v^	0.86	2.07	2.898 (5)	161
N4—H4A···Cl1^vi^	0.86	2.71	3.512 (3)	155
N4—H4B···Cl1^ii^	0.86	2.59	3.433 (3)	167

Symmetry codes: (ii) *x*, *y*+1, *z*; (iii) −*x*+1/2, −*y*+3/2, −*z*; (iv) −*x*+1/2, *y*−1/2, −*z*+1/2; (v) *x*, −*y*, *z*+1/2; (vi) −*x*+1/2, *y*+1/2, −*z*+1/2.
